# Elucidation of the liver pathophysiology of COVID-19 patients using liver-on-a-chips

**DOI:** 10.1093/pnasnexus/pgad029

**Published:** 2023-03-07

**Authors:** Sayaka Deguchi, Kaori Kosugi, Rina Hashimoto, Ayaka Sakamoto, Masaki Yamamoto, Rafal P Krol, Peter Gee, Ryosuke Negoro, Takeshi Noda, Takuya Yamamoto, Yu-suke Torisawa, Miki Nagao, Kazuo Takayama

**Affiliations:** Center for iPS Cell Research and Application (CiRA), Kyoto University, Kyoto 606-8507, Japan; Department of Medical Science, Graduate School of Medicine, Kyoto University, Kyoto 606-8507, Japan; Center for iPS Cell Research and Application (CiRA), Kyoto University, Kyoto 606-8507, Japan; Center for iPS Cell Research and Application (CiRA), Kyoto University, Kyoto 606-8507, Japan; Center for iPS Cell Research and Application (CiRA), Kyoto University, Kyoto 606-8507, Japan; Department of Clinical Laboratory Medicine, Graduate School of Medicine, Kyoto University, Kyoto 606-8507, Japan; CiRA Foundation, Research and Development Center, Kyoto 606-8397, Japan; MaxCyte, Inc., Gaithersburg, MD 20878, USA; Laboratory of Molecular Pharmacokinetics, College of Pharmaceutical Sciences, Ritsumeikan University, Kusatsu 525-8577, Japan; Laboratory of Ultrastructural Virology, Institute for Frontier Life and Medical Sciences, Kyoto University, Kyoto 606-8507, Japan; CREST, Japan Science and Technology Agency (JST), Kawaguchi 332-0012, Japan; Center for iPS Cell Research and Application (CiRA), Kyoto University, Kyoto 606-8507, Japan; Institute for the Advanced Study of Human Biology (WPI-ASHBi), Kyoto University, Kyoto 606-8501, Japan; Medical-risk Avoidance based on iPS Cells Team, RIKEN Center for Advanced Intelligence Project (AIP), Kyoto 606-8507, Japan; Department of Micro Engineering, Kyoto University, Kyoto 615-8540, Japan; Department of Clinical Laboratory Medicine, Graduate School of Medicine, Kyoto University, Kyoto 606-8507, Japan; Center for iPS Cell Research and Application (CiRA), Kyoto University, Kyoto 606-8507, Japan; AMED-CREST, Japan Agency for Medical Research and Development (AMED), Tokyo 100-0004, Japan

**Keywords:** SARS-CoV-2, COVID-19, organs-on-a-chip, liver-on-a-chip, Remdesivir, Baricitinib

## Abstract

SARS-CoV-2 induces severe organ damage not only in the lung but also in the liver, heart, kidney, and intestine. It is known that COVID-19 severity correlates with liver dysfunction, but few studies have investigated the liver pathophysiology in COVID-19 patients. Here, we elucidated liver pathophysiology in COVID-19 patients using organs-on-a-chip technology and clinical analyses. First, we developed liver-on-a-chip (LoC) which recapitulating hepatic functions around the intrahepatic bile duct and blood vessel. We found that hepatic dysfunctions, but not hepatobiliary diseases, were strongly induced by SARS-CoV-2 infection. Next, we evaluated the therapeutic effects of COVID-19 drugs to inhibit viral replication and recover hepatic dysfunctions, and found that the combination of anti-viral and immunosuppressive drugs (Remdesivir and Baricitinib) is effective to treat hepatic dysfunctions caused by SARS-CoV-2 infection. Finally, we analyzed the sera obtained from COVID-19 patients, and revealed that COVID-19 patients, who were positive for serum viral RNA, are likely to become severe and develop hepatic dysfunctions, as compared with COVID-19 patients who were negative for serum viral RNA. We succeeded in modeling the liver pathophysiology of COVID-19 patients using LoC technology and clinical samples.

**Significance Statement**

Using liver-on-a-chip that included an intrahepatic bile duct (ibd-LoC) or blood vessel (bv-LoC), it was found that SARS-CoV-2 induced more severe hepatic damages in bv-LoCs than in ibd-LoCs. Consistently, in serum samples obtained from severe COVID-19 patients, the levels of hepatic enzymes were elevated, and viral RNA was detected. These results suggested that the pathophysiology of COVID-19 patients could be revealed by using LoC technology and clinical samples.

## Introduction

Severe acute respiratory syndrome coronavirus 2 (SARS-CoV-2) is the causative virus of Coronavirus disease 2019 (COVID-19). SARS-CoV-2 induces multi-organ damage by infecting not only the respiratory tract but also other organs. In addition to respiratory diseases, extrapulmonary diseases, including liver, heart, kidney, intestine, or neuron dysfunctions, are observed in COVID-19 patients (1). Because COVID-19 severity is known to correlate with liver dysfunction, it is important to understand the liver pathophysiology of COVID-19 patients (2). SARS-CoV-2 spike protein was detected in liver autopsy samples from patients who died of COVID-19, suggesting that SARS-CoV-2 may infect the liver (3). It is possible to isolate SARS-CoV-2 from liver autopsy samples as well as from lung autopsy samples of COVID-19 patients (4). In severe COVID-19 patients, it is known that multiple organ failure is observed two to three weeks after the onset, but recent important cohorts of patients with COVID-19 requiring hospitalization have demonstrated that liver injury markers, alanine aminotransferase (ALT) and aspartate aminotransferase (AST), were increased sharply only 1 day after admission (4). A recent study showed that zone-specific damage is observed in COVID-19 patients. In the hepatic region around the blood vessels, inflammatory responses were induced, and the portal vein was enlarged (5, 6). On the other hand, significant histological damage was not observed in the hepatic region around the bile ducts (6). However, the process and mechanism of the liver lesions caused by SARS-CoV-2 infection have not been fully elucidated. Therefore, a liver model that can elucidate the liver pathophysiology in COVID-19 patients has to be developed.

We recently generated liver-on-a-chips (LoCs) using a microfluidic device with two parallel microchannels for pharmaceutical research (7, 8). Using LoC technology, we can culture liver cells in three-dimensional (3D) conditions and expose them to physiological stresses, such as mechanical stretch or fluid flow. Here, we refined our LoCs to include either an intrahepatic bile duct (ibd-LoC) or blood vessel (bv-LoC) to clarify the pathophysiology of the liver around the bile ducts or blood vessels, respectively. Using ibd- and bv-LoCs, we recapitulated and elucidated the liver pathophysiology of COVID-19 patients and evaluated the therapeutic efficacy of COVID-19 drugs.

Technical advances in in vitro cell models, including organs-on-a-chips, have made it possible to accurately reproduce pathophysiologies in vitro. However, it is still difficult to clarify the whole picture of pathophysiology using only these models. Thus, an analysis of clinical samples is also needed. We have recently clarified the respiratory pathophysiology of COVID-19 patients using airway-on-a-chips and clinical samples (9). We believe that liver damage in severe COVID-19 patients is the next important issue to be resolved after respiratory damage, thus we attempted to recapitulate the liver pathophysiology of COVID-19 patients by simultaneously performing SARS-CoV-2 experiments using LoCs and analyzing clinical specimens of COVID-19 patients.

## Results

### Generation of ibd-LoCs and bv-LoCs

**Fig. 1.**
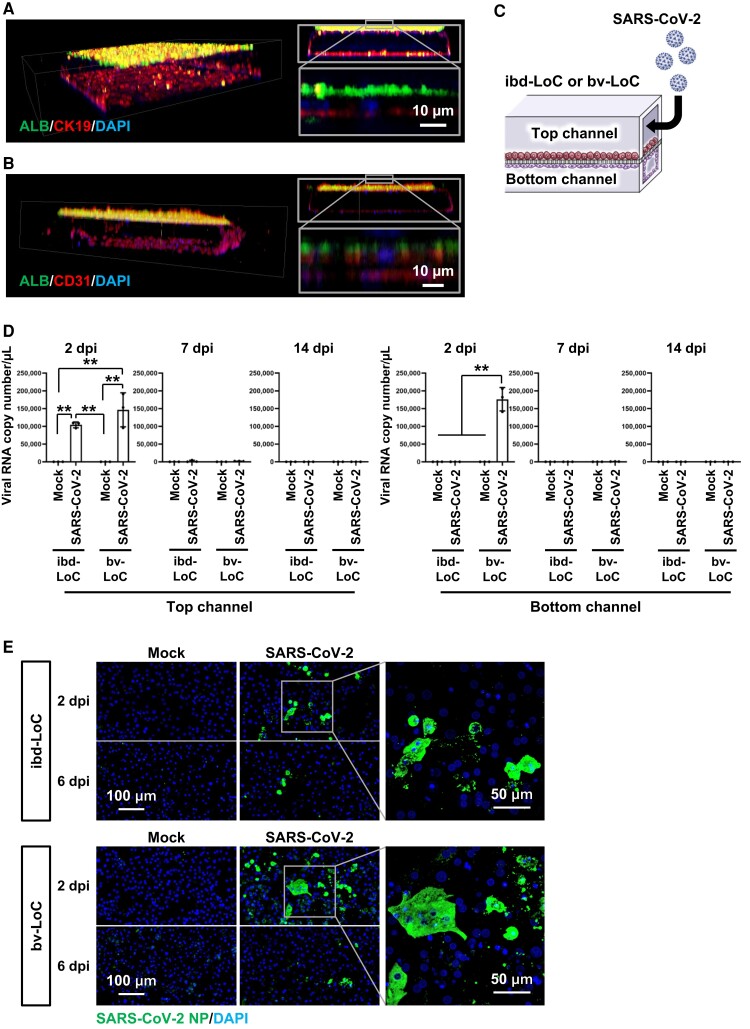
Generation of ibd- and bv-LoCs. (A, B) 3D images of the ibd- and bv-LoCs. Immunostaining analysis of ALB and CK19 in the ibd-LoC (A) and of ALB and CD31 in the bv-LoC (B) were performed. Nuclei were counterstained with DAPI. (C) Schematic overview of the SARS-CoV-2 infection experiment using ibd- and bv-LoCs. SARS-CoV-2 (0.1 MOI) was injected into the top channel. (D) At 2, 7, and 14 dpi, the viral RNA copy number in the cell culture supernatant was measured by quantitative real time-PCR (qPCR). Two-way ANOVA followed by Tukey's post hoc test (**P < 0.01) (E) At 2 and 6 dpi, immunostaining analysis of SARS-CoV-2 NP in hepatocytes of ibd- and bv-LoCs was performed. Nuclei were counterstained with DAPI. Data are representative of three independent experiments and are represented as the means ± SD (*n* = 3, technical replicates).

To reproduce the hepatic region around the bile duct, we generated ibd-LoCs that contain hepatocytes and cholangiocytes in the microfluidic devices. We also generated bv-LoCs that contain hepatocytes and endothelial cells in the microfluidic devices to reproduce the hepatic region around blood vessels. Our microfluidic device has two microchannels that are separated by two PET membranes (Fig. S1A). Because our microfluidic device has two PET membranes, cells in the top and bottom channels can be easily collected separately. The ibd-LoC was developed by culturing human hepatocytes and cholangiocytes in the top and bottom channels of the device, respectively (Fig. S1B). In contrast, the bv-LoC was developed by culturing human hepatocytes and endothelial cells in the top and bottom channels, respectively. Immunostaining analysis of albumin (ALB: hepatocyte marker) and cytokeratin 19 (CK19; cholangiocyte marker) in the ibd-LoC revealed that cholangiocytes form a tubular structure in the bottom channel with a hepatocyte monolayer adjacent to the structure (Fig. 1A). Immunostaining analysis of ALB and CD31 (endothelial marker) in the bv-LoC revealed that endothelial cells form a tubular structure in the bottom channel, with the hepatocyte monolayer adjacent to the structure (Fig. 1B). Note that we have confirmed that ibd- and bv-LoCs can be stably cultured for at least two weeks (Fig. S1C–F). Next, we examined whether the selective transport activity of bile and blood components can be reproduced using ibd- and bv-LoCs (Fig. S1G). In the human liver, bile or blood components synthesized in hepatocytes are transported into the bile ducts or blood vessels, respectively. The concentration of bile components (bile acids and direct bilirubin) in the bottom channel (bile duct channel) of ibd-LoCs was higher than that in the bottom channel (blood vessel channel) of bv-LoCs. On the other hand, the concentration of blood component (albumin) in the bottom channel (blood vessel channel) of bv-LoCs was higher than that in the bottom channel (bile duct channel) of ibd-LoCs. These results suggest that we succeeded in generating LoCs with intrahepatic bile duct and blood vessel.

### SARS-CoV-2-infected hepatocytes in ibd- and bv-LoCs

To elucidate the liver pathophysiology in COVID-19 patients, ibd- and bv-LoCs were infected with SARS-CoV-2. Before performing infection experiments using ibd- and bv-LoCs, component cells of ibd- and bv-LoCs, including hepatocytes, cholangiocytes, and endothelial cells, were separately infected with SARS-CoV-2 (Figs. S2A and B). The SARS-CoV-2 infection efficiency in human hepatocytes was higher than that in cholangiocytes. SARS-CoV-2 did not infect endothelial cells. Then we examined the gene expression levels of two essential host factors for SARS-CoV-2 infection, *angiotensin-converting enzyme2 (ACE2)* and *transmembrane serine protease 2 (TMPRSS2)*, in the hepatocytes of the ibd- and bv-LoCs and found their expression levels in hepatocytes were similar between both LoCs (Fig. S2C). Therefore, it is expected that hepatocytes are the main target cells of SARS-CoV-2 in ibd- and bv-LoCs.

In this study, medium containing SARS-CoV-2 was infected into the top channel of the ibd- and bv-LoCs to investigate the effects of the virus on the liver (Fig. 1C). At 2 days post-infection (dpi), viral RNA was detected in the cell culture supernatant collected from the top channels (Figs. 1D and S3A). On the other hand, at 7 and 14 dpi, viral RNA was hardly detected in the cell culture supernatant collected from the top channels. Viral RNA was also detected in the cell culture supernatant collected from the bottom channel of bv-LoCs but not of ibd-LoCs (Figs. 1D and S3B). This result suggests that SARS-CoV-2 could enter the blood vessel channel but not the intrahepatic bile duct channel. Immunostaining analysis of SARS-CoV-2 nucleocapsid protein (NP) in hepatocytes of ibd-LoCs was performed (Fig. 1E). At 2 dpi, NP-positive hepatocytes were observed, but hardly at 6 dpi. These results indicate that SARS-CoV-2 can infect hepatocytes in both ibd- and bv-LoCs, but most of the virus is excluded at 6 dpi.

### Expression levels of IFN-related genes were increased in hepatocytes in bv-LoC, but not in ibd-LoC, by SARS-CoV-2 infection

**Fig. 2.**
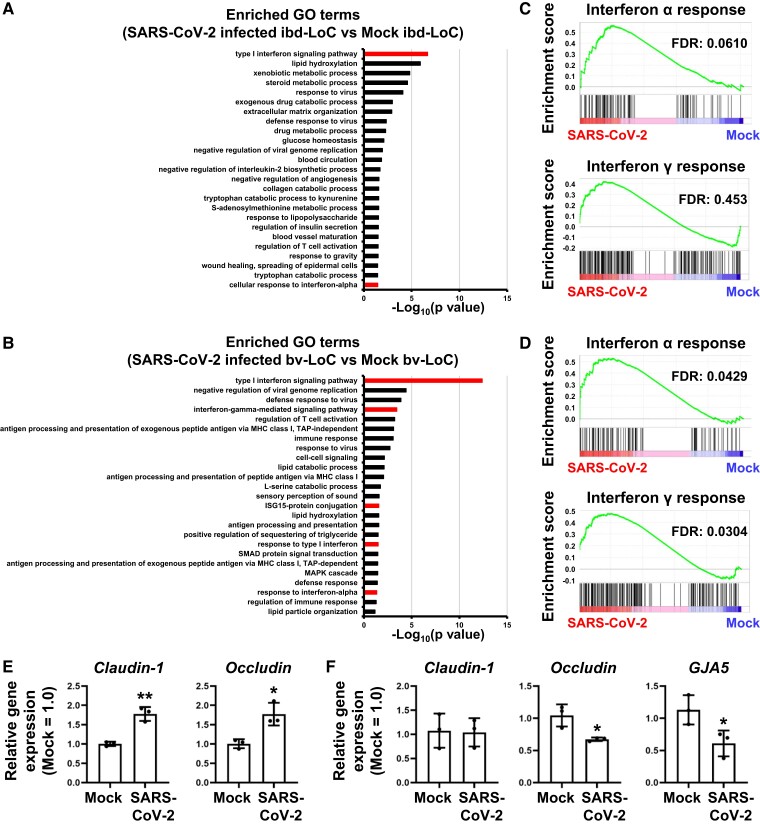
IFN-related gene expressions and endothelial barrier disruption was induced in bv-LoC by SARS-CoV-2 infection. The ibd- and bv-LoCs were infected with 0.1 MOI SARS-CoV-2. (A-D) RNA-seq analysis of mock or SARS-CoV-2-infected hepatocytes in ibd- and bv-LoCs at 4 dpi. (A and B) A GO enrichment analysis of mock versus SARS-CoV-2-infected hepatocytes in (A) ibd- and (B) bv-LoCs. Interferon-related terms are shown in red bars. (C-D) A GSEA using hallmark gene sets of mock versus SARS-CoV-2-infected hepatocytes in (C) ibd- and (D) bv-LoCs. (E and F) The gene expression levels of junction markers in cholangiocytes in ibd-LoCs (E) or endothelial cells in bv-LoCs (F) were measured by RT-qPCR. The gene expression levels in mock were taken as 1.0. Unpaired two-tailed Student's *t*-test (*P < 0.05, **P < 0.01). Data are representative of three independent experiments and are represented as the means ± SD (*n* = 3, technical replicates).

RNA-sequencing analysis was performed to analyze the gene expression profile changes in hepatocytes of ibd- and bv-LoCs induced by SARS-CoV-2 infection. The expression levels of 99 and 143 genes in hepatocytes of ibd- and bv-LoCs, respectively, were significantly up-regulated by SARS-CoV-2 infection (more than two-fold) (Fig. S4). Gene Ontology (GO) enrichment analysis of these differentially expressed genes showed that GO terms with interferon (IFN)-related genes were enriched in hepatocytes of ibd- and bv-LoCs by the viral infection (Fig. 2A and B). The frequency of IFN-related terms in hepatocytes of bv-LoCs was higher than in hepatocytes of ibd-LoCs. In addition, a gene set enrichment analysis (GSEA) was performed (Fig. 2C and D). The hallmark gene sets of IFN α or γ responses were significantly enriched by SARS-CoV-2 infection in hepatocytes of bv-LoCs (Fig. 2D). The induction of the gene expression levels of *IFNA1*, *IFNB1*, *ISG15*, and *MxA* in hepatocytes of bv-LoCs by SARS-CoV-2 infection was higher than in hepatocytes of ibd-LoCs (Fig. S5A and B). These results indicated that the IFN-related genes were strongly induced by SARS-CoV-2 infection in hepatocytes of bv-LoCs but not of ibd-LoCs.

### Endothelial barrier disruption was occurred in bv-LoC by SARS-CoV-2 infection

Next, we examined the epithelial and endothelial barrier function after the SARS-CoV-2 infection. The expression levels of several cell–cell junction-related genes (*claudin-1*, *occludin*, and *gap junction protein alpha* 5 (*GJA5)*) in cholangiocytes and endothelial cells of ibd- and bv-LoCs, respectively, were examined (Fig. 2E and F). The expression levels of these genes in endothelial cells were decreased by SARS-CoV-2 infection, suggesting that SARS-CoV-2 infection compromises endothelial barrier function. The leakage of virus in the blood vessel channel observed in Fig. 1D might be due to this endothelial barrier disruption. Then, we examined whether the endothelial barrier disruption was caused by the exposure to SARS-CoV-2 or factors released from infected hepatocytes. The virus leakage in the bottom channel of bv-LoCs cannot be observed in the absence of infected hepatocytes (Fig. S5C). These results suggest that the factors released from infected hepatocytes contribute to the vascular endothelial barrier disruption in bv-LoC.

### Liver dysfunctions were caused in hepatocytes of bv-LoCs by SARS-CoV-2 infection

It remains unclear whether the liver dysfunctions observed in COVID-19 patients are caused by direct effects of SARS-CoV-2 infection or indirect effects mediated by cytokines produced from other organs. Therefore, we investigated the liver pathophysiology in COVID-19 patients using ibd- and bv-LoCs.

**Fig. 3.**
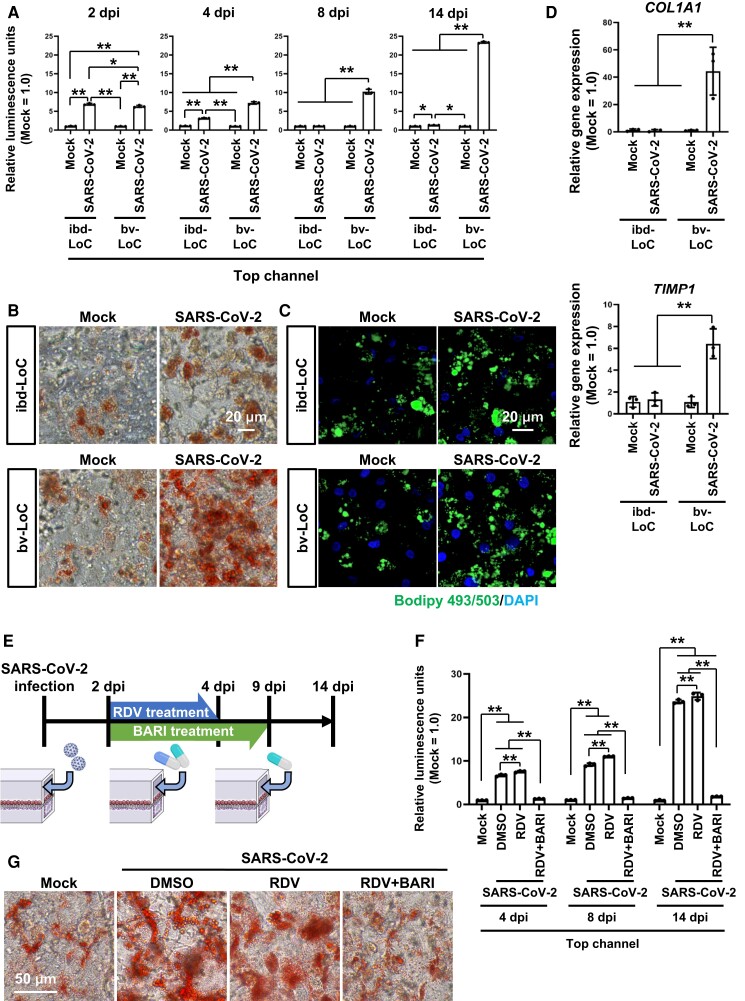
The combination of RDV and BARI suppresses both SARS-CoV-2 replication and liver damage. The ibd- and bv-LoCs were infected with 0.1 MOI SARS-CoV-2. (A) At 2, 4, 8, and 14 dpi, LDH release in the cell culture supernatant was measured. The LDH release in mock was taken as 1.0. Two-way ANOVA followed by Tukey's post hoc test (*P < 0.05, **P < 0.01). (B) At 14 dpi, lipid droplets in hepatocytes in ibd- and bv-LoCs were stained with Oil red O. (C) At 14 dpi, lipid droplets in hepatocytes in ibd- and bv-LoCs were stained with BODIPY 493/503. Nuclei were counterstained with DAPI. (D) At 14 dpi, the gene expression levels of *COL1A1* and *TIMP1* were measured by RT-qPCR. The gene expression levels in mock were taken as 1.0. Two-way ANOVA followed by Tukey's post hoc test (**P < 0.01). (E) Schematic overview showing the protocol for the SARS-CoV-2 infection and treatment with RDV and BARI. (F) At 4, 8, and 14 dpi, LDH release in the cell culture supernatant in the top channel was measured. Two-way ANOVA followed by Tukey's post hoc test (**P < 0.01). (G) At 14 dpi, lipid droplets in hepatocytes in bv-LoCs were stained with Oil red O. Data are representative of three independent experiments and are represented as the means ± SD (*n* = 3, technical replicates).

After SARS-CoV-2 infection, the amount of lactate dehydrogenase (LDH), which was released from damaged cells, in ibd- and bv-LoCs was evaluated (Figs. 3A and S6A). LDH release in bv-LoCs was increased by SARS-CoV-2 infection at 2, 4, 8, and 14 dpi. The LDH release in ibd-LoCs was moderately increased by SARS-CoV-2 infection at 2, 4, 8, and 14 dpi. Next, lipid droplets in hepatocytes of ibd- and bv-LoCs were stained using Oil Red O (Fig. 3B) and BODIPY 493/503 (Fig. 3C). At 14 dpi, lipid droplets were accumulated in the hepatocytes of SARS-CoV-2-infected bv-LoCs. Then, the gene expression levels of liver fibrosis markers were evaluated (Fig. 3D). At 14 dpi, the gene expression levels of the fibrosis markers *COL1A1* and *TIMP1* in the hepatocytes of SARS-CoV-2-infected bv-LoCs were significantly increased. We also compared the liver damage using three SARS-CoV-2 strains (B.1.1.214, B.1.617.2, and B.1.1.529), and found that liver damage was moderate in SARS-CoV-2 B.1.1.529 (Omicron)-infected bv-LoC, compared with the other variant-infected bv-LoCs (Fig. S6B–D). Taken together, these results suggest that SARS-CoV-2 infection directly causes liver dysfunctions.

### RDV and MPV treatment suppressed SARS-CoV-2 replication but did not reduce liver damage

Remdesivir (RDV) and molnupiravir (MPV) are approved COVID-19 drugs (10–13). Both inhibit viral RNA-dependent RNA polymerase activity. Here, we examined whether RDV or MPV treatment could inhibit viral infection and recover liver function using bv-LoCs. First, we examined whether our device is suitable for analyzing RDV and MPV. Our device is made of an elastomer, polydimethylsiloxane (PDMS). PDMS easily absorbs some small hydrophobic molecules, but we previously found that compounds with low S + log D values, like RDV and MPV, are hardly absorbed (Fig. S7A) (7). We injected the medium containing RDV or MPV into the top and bottom channels of the PDMS-based device and evaluated the concentration of the drugs at 1, 2, and 4 h after the injection (Fig. S7B). As expected, RDV and MPV were not absorbed into the PDMS-based device. We also evaluated the hepatotoxicity of RDV and MPV (Fig. S8A). Because 2 μM RDV or 10 μM MPV treatment did not show a cytotoxic effect on hepatocytes, we performed the following infection experiments under these concentrations.

The anti-viral effects of RDV and MPV were examined using bv-LoCs. After SARS-CoV-2 infection, RDV- or MPV-containing medium was injected into the top channel of bv-LoCs at 2–5 dpi (Fig. S8B). A viral genome was detected in the supernatant of bv-LoCs before the drug treatment (2 dpi, Fig. S8C), but it was hardly detected afterward (4 or 8 dpi, Fig. S8D). To examine whether the viral infection-mediated cell toxicity can be reduced by RDV or MPV treatment, the amount of LDH was evaluated (Fig. S8E and F). Interestingly, the amount of LDH released in the supernatant was not reduced by the treatment (Fig. S8F). These results suggest that RDV or MPV treatment can inhibit SARS-CoV-2 infection but not the viral infection-mediated cell toxicity.

### The combination of RDV and BARI suppressed both SARS-CoV-2 replication and liver damage

In a recent clinical report, the combination of RDV and baricitinib (BARI) was superior to RDV alone at accelerating the improved clinical status of COVID-19 patients (14). BARI is a selective inhibitor of Janus kinase (JAK) 1 and 2, and BARI treatment suppresses interleukin-6 or IFN-γ secretion in COVID-19 patients (15). Therefore, we investigated the therapeutic effects of RDV and BARI using bv-LoCs.

First, we examined whether the PDMS-based device is suitable for analyzing BARI. Again, the S + log D value of BARI is low (Fig. S7A). We injected the medium containing BARI into the top and bottom channels of the PDMS-based device and evaluated the concentration of BARI at 1, 2, and 4 h after the drug injection. As expected, BARI was not absorbed into the PDMS-based device (Fig. S7B). Then we examined the hepatotoxicity of BARI (Fig. S9A). Because 1 μM BARI alone or the combination of 2 μM RDV and 1 μM BARI did not show a cytotoxic effect on hepatocytes, we performed the following infection experiments under this concentration.

After the SARS-CoV-2 infection, medium containing RDV alone or RDV and BARI was injected into the top channel of bv-LoCs at 2–4 dpi (Fig. 3E). Medium containing BARI alone was added at 4–9 dpi. As above, the viral genome was detected in the supernatant of bv-LoCs before the drug treatment (2 dpi, Fig. S9B) but hardly afterward (4 and 8 dpi, Fig. S9C). To examine whether the viral infection-mediated cell toxicity can be reduced by RDV and BARI treatment, the amount of LDH was evaluated (Figs. 3F, S9D and E). Importantly, the amount of LDH in infected bv-LoCs was decreased by RDV and BARI treatment but not by RDV alone treatment (Figs. 3F and S9E). Consistently, Oil Red O staining showed that the accumulation of lipid droplets in hepatocytes of infected bv-LoCs was reduced by RDV and BARI treatment (Fig. 3G). These results suggest that the combination of RDV and BARI inhibits SARS-CoV-2 infection and recovers liver function.

### Comparison of data from LoCs and clinical data of COVID-19 patients

**Fig. 4.**
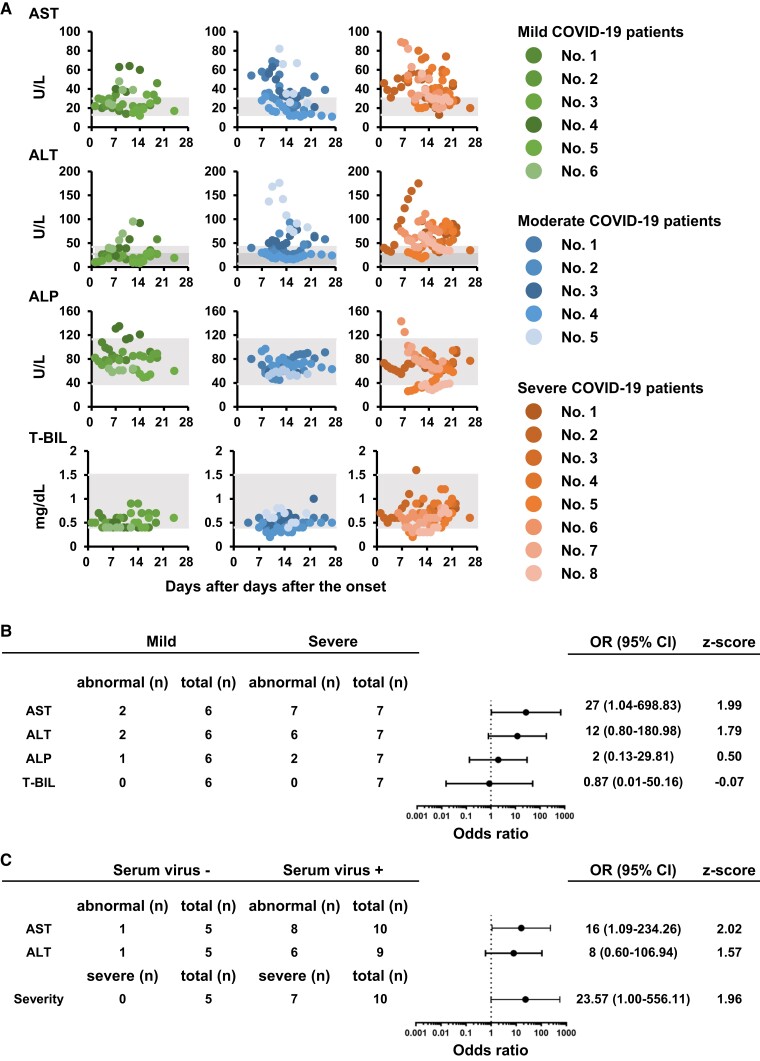
Comparison of the results obtained from LoCs and clinical data of COVID-19 patients. (A) The temporal change in serum levels of AST, ALT, ALP, and T-BIL after the onset of symptoms in patients with mild (left), moderate (middle) or severe (right) COVID-19. The gray areas represent the reference range for normal values. (B) Odds ratios and 95% CI for liver function markers in COVID-19 patients. The number of patients who showed abnormal values (higher than normal range) were counted using data in Figure A. (C) Odds ratios and 95% CI for liver function markers and serum virus in COVID-19 patients. The number of patients who showed abnormal values (higher than normal range) were counted using data in Table S1.

Finally, the blood test data and serum viral genome copy number in COVID-19 patients were compared with the LoC results. Our in vitro data detected hepatic dysfunctions in bv-LoCs but no hepatobiliary damage in ibd-LoCs. Thus, the serum values of hepatic dysfunction markers (AST and ALT) and hepatobiliary disease markers (alkaline phosphatase (ALP) and total bilirubin (T-BIL)) in mild, moderate, and severe COVID-19 patients (without active liver disease) were examined (Figs. 4A, S10A and B). The gray areas represent the reference range for normal values. We calculated the average values of each marker within 14 days after the onset and compared them with normal values (Fig. 4B). The AST and ALT values were elevated in severe COVID-19 patients, as compared with those in mild COVID-19 patients (Fig. S10A). The probabilities of elevated AST or ALT in severe COVID-19 patients were higher than those in mild COVID-19 patients (AST: odds ratio = 27, 95% confidence intervals (CI) = 1.04–698.83; ALT: odds ratio = 12, 95% CI = 0.80–180.98) (Fig. 4B). On the other hand, the probabilities of abnormal ALP and T-BIL in mild and severe COVID-19 patients were similar to each other. These results suggest that hepatic dysfunction but not hepatobiliary disease was induced in severe COVID-19 patients. The findings from the clinical samples analysis are consistent with the phenomena observed in our in vitro data.

Because our in vitro analysis demonstrated that SARS-CoV-2 breached the endothelial barrier and leaked into the blood vessel channel, we examined whether viral RNA is in the sera of COVID-19 patients. Although viral RNA was not detected in serum collected more than 7 days after the onset, it was detected in some sera (10 patients/15 patients) collected within 7 days after the onset (Table S1). We calculated the average AST or ALT values in 15 patients. Interestingly, the probabilities of elevated AST or ALT in COVID-19 patients who were positive for serum viral RNA were higher than those in COVID-19 patients who were negative for serum viral RNA (AST: odds ratio = 16, 95% CI = 1.09–234.26; ALT: odds ratio = 8, 95% CI = 0.60–106.94) (Fig. 4C). Additionally, the probability of serum virus-positive in severe COVID-19 patients was higher than in mild and moderate COVID-19 patients (odds ratio = 23.57; 95% CI = 1.00–556.11). Thus, we propose that COVID-19 patients in whom serum virus is detected within 7 days of onset are likely to become severe and develop liver damage.

## Discussion

SARS-CoV-2 infection caused endothelial barrier dysfunction only in bv-LoCs. Additionally, liver dysfunctions were observed in bv-LoCs but not in ibd-LoCs. The reason for the different results between the two LoC models could be the properties of their hepatocytes. We also evaluated the therapeutic effects of several COVID-19 drugs using bv-LoCs and found that the combination of RDV and BARI was effective at recovering hepatic functions in infected bv-LoCs, suggesting a combination of anti-viral drugs and immunoregulating drugs is effective at treating organ dysfunctions in COVID-19 patients. Future experiments using organs-on-a-chip models of other organs are needed to test this hypothesis.

In SARS-CoV-2-infected bv-LoCs, endothelial barrier function was decreased. Such endothelial barrier disruption was also reported in SARS-CoV-2-infected airway-, lung- and gut-on-a-chip (9, 16, 17). However, since the mechanism of endothelial barrier damage may differ depending on the organ type, analysis using individual organ models will be essential. It is also reported that endothelial dysfunctions can be caused by cytokines induced by the infecting virus, such as SARS-CoV-2, influenza virus, and lymphocytic choriomeningitis virus (18, 19). To perform a more accurate investigation of endothelial barrier disruption by cytokines, it is essential to develop a model that includes immune cells that produce cytokines in response to viral infection. Although the bv- and ibd-LoCs we developed do not contain immune cells, we should be able to build models that accurately reflect in vivo conditions by using immune cells in the future.

Besides the disadvantage that our bv- and ibd-LoCs do not contain immune cells, these models have another important limitation. Because we generated bv-LoCs and ibd-LoCs using separate microfluidic devices, we cannot accurately reproduce liver zonation. In vivo hepatocyte functions are known to be heterogeneous depending on their zonality. To recapitulate the liver zonation using LoCs, it is required to generate LoCs which culture hepatocytes, cholangiocytes, and endothelial cells in the same microfluidic device. Additionally, if we obtain culturable endothelial cells of the portal vein, central vein, and hepatic artery, it would be possible to recapitulate the zonal heterogeneity in more detail.

In addition to liver dysfunction, dysfunctions in other organs are observed in COVID-19 patients. By using organs-on-a-chip technology, we can connect multiple organ models (20, 21). For example, by connecting LoC and lung-on-a-chip, we can analyze the liver dysfunction caused by cytokines produced from the infected lung. Overall, organs-on-a-chip technology could help analyze the whole-body response in COVID-19 patients.

In this study, we used a clinical approach in addition to a LoC technology to elucidate the liver pathophysiology of COVID-19 patients. In vitro experiments using LoCs suggested that SARS-CoV-2 infection causes hepatic dysfunctions rather than hepatobiliary damage. This result was supported by a blood test in COVID-19 patients. The same in vitro experiments also suggested that SARS-CoV-2 invades blood vessels where the hepatocellular injury occurs. This observation was supported by quantifying the viral copy number in the sera of COVID-19 patients. Therefore, by combining LoC technology and a clinical approach, we demonstrated that the liver pathophysiology of COVID-19 patients could be clarified in detail.

## Materials and methods

### Cell culture

Before seeding cholangiocytes or endothelial cells, a bottom channel of the PDMS device was pre-coated with fibronectin (1 μg/mL, Sigma-Aldrich). Cholangiocytes (HuCCT1 cells, JCRB0425, JCRB Cell Bank) or endothelial cells (Human Umbilical Vein Endothelial Cells: HUVECs, Lonza) were suspended at 5 × 10^6^ cells/mL in RPMI-1640 medium containing 1×GlutaMAX (Thermo Fisher Scientific) and 10% fetal bovine serum (FBS) or EGM-2 Endothelial Cell Growth Medium-2 BulletKit (Lonza), respectively. Ten μL suspension medium was injected into the fibronectin-coated bottom channel. Then, the device was turned upside down and incubated for 1 h. After the incubation, the device was turned over, and the medium was injected into the bottom channel.

After 4 days, human hepatocytes (HUCPI, Lonza) were seeded into the top channel. Before seeding hepatocytes, the PDMS devices were pre-coated with Collagen I solution (30 μg/mL, Corning). The vials of human hepatocytes were rapidly thawed in a shaking water bath at 37°C. The contents of each vial were emptied into pre-warmed Cryopreserved Hepatocyte Recovery Medium (Thermo Fisher Scientific), and the suspension was centrifuged at 1,200 rpm for 5 min at room temperature. Hepatocytes were suspended at 5 × 10^6^ cells/mL in Hepatocyte Culture Medium BulletKit (HCM, Lonza) containing 10% FBS. Ten μL suspension medium was injected into a Collagen Type I (Corning)-coated PDMS device. After 1 h, the medium was added into the top and bottom channels.

### Quantitative real time-PCR

ISOGEN (NIPPON GENE) was used to isolate total RNA from the cells. A Superscript VILO cDNA Synthesis Kit (Thermo Fisher Scientific) was used to synthesize cDNA from the isolated total RNA. Quantitative real time-PCR (qPCR) was performed with SYBR Green PCR Master Mix (Thermo Fisher Scientific) using a StepOnePlus qPCR system (Thermo Fisher Scientific). The 2^−ΔΔCT^ method was adopted for the relative quantitation of the target mRNA levels. The values of the target genes were normalized by those of the housekeeping gene, *glyceraldehyde 3-phosphate dehydrogenase* (*GAPDH*). The PCR primer sequences are summarized in Table S2.

### Immunostaining analysis

The ibd- and bv-LoCs were fixed with 4% paraformaldehyde in PBS for 15 min. After blocking the cells with PBS containing 10% FBS, 1% bovine serum albumin, and 0.2% Triton X-100 at room temperature for 45 min, the cells were incubated with a primary antibody at room temperature for 2 h and then with a secondary antibody at room temperature for 1 h. All antibodies used in this study are described in Table S3.

### RDV, MPV, and BARI absorption into the PDMS device

Medium containing 10 μM of RDV, MPV, or BARI was injected into the PDMS device (200 μL medium/channel). Half of the medium was collected at 1, 2, or 4 h after the drug treatment. When collecting the supernatant, the same amount of culture medium containing the substrate was added. The collected supernatant was mixed with the same volume of acetonitrile. Samples were filtrated with Cosmonice Filter W of a pore size of 0.45 µm and then analyzed by HPLC to measure the concentration of RDV, MPV, or BARI according to a standard curve. HPLC analysis was performed using a LO-20AD SPD + RF (DGU-20A, LC-20AD, RF-20A xs, SIL-20AC, CBM-20A, SPD-20A, CTO-20AC; Shimadzu). The HPLC methods are summarized in Table S4.

## Supplementary Material

pgad029_Supplementary_Data

## Data Availability

Raw data concerning RNA-seq analysis were submitted under Gene Expression Omnibus (GEO) accession number GSE193330.
