# Supplementary material for: Elucidation of the liver pathophysiology of COVID-19 patients using liver-on-a-chips
Source: PNAS Nexus. 2023 Mar 7;2(3):pgad029. doi: 10.1093/pnasnexus/pgad029 (PMC9991504; doi:10.1093/pnasnexus/pgad029)
Supplement: pgad029_Supplementary_Data [file pgad029_supplementary_data.pdf]

Supplemental data

Elucidation of the liver pathophysiology of COVID-19 patients using liver-on-a-chips

Authors/Affiliations
Sayaka Deguchi[1,2]
Kaori Kosugi[1]
Rina Hashimoto[1]
Ayaka Sakamoto[1]
Masaki Yamamoto[3]
Rafal P. Krol[4]
Peter Gee[5]
Ryosuke Negoro[6]
Takeshi Noda[7,8]
Takuya Yamamoto[1,9,10]
Yu-suke Torisawa[11]
Miki Nagao[3]
Kazuo Takayama[1,12,*]

[1] Center for iPS Cell Research and Application (CiRA), Kyoto University, Kyoto 606-8507, Japan
[2] Department of Medical Science, Graduate School of Medicine, Kyoto University, Kyoto 606-8507, Japan
[3] Department of Clinical Laboratory Medicine, Graduate School of Medicine, Kyoto University, Kyoto 606-8507, Japan
[4] CiRA Foundation, Research and Development Center, Kyoto 606-8397, Japan
[5] MaxCyte, Inc., Gaithersburg, MD 20878, United States of America
[6] Laboratory of Molecular Pharmacokinetics, College of Pharmaceutical Sciences, Ritsumeikan University, Noji-Higashi, Kusatsu 525-8577, Japan
[7] Laboratory of Ultrastructural Virology, Institute for Frontier Life and Medical Sciences, Kyoto University, Kyoto 606-8507, Japan
[8] CREST, Japan Science and Technology Agency (JST), Kawaguchi 332-0012, Japan
[9] Institute for the Advanced Study of Human Biology (WPI-ASHBi), Kyoto University, Kyoto 606-8501 Japan
[10] Medical-risk Avoidance based on iPS Cells Team, RIKEN Center for Advanced

Intelligence Project (AIP), Kyoto 606-8507, Japan
[11] Department of Micro Engineering, Kyoto University, Kyoto 615-8540, Japan
[12] AMED-CREST, Japan Agency for Medical Research and Development (AMED), Tokyo 100-0004, Japan

***Corresponding author**
Dr. Kazuo Takayama
Center for iPS Cell Research and Application (CiRA), Kyoto University, Shogoin Kawaharacho 53, Sakyo-ku, Kyoto 606-8507, Japan.
Phone: +81-75-366-7362, FAX: +81-75-366-7098
E-mail: kazuo.takayama@cira.kyoto-u.ac.jp

Supplementary materials and methods

Fabrication of microfluidic devices

The microfluidic device consists of two layers of microchannels separated by two semipermeable membranes (**Fig. 1A**). The microchannel layers were fabricated from PDMS using a soft lithographic method (1). PDMS prepolymer (Sylgard 184, Dow Corning) at a ratio of 10:1 base to curing agent was cast against a mold composed of SU-8 2150 (MicroChem) patterns formed on a silicon wafer. The cross-sectional size of the microchannels was 1 mm in width and 300 μm in height. To introduce solutions into the microchannels, access holes were punched through the PDMS using a 6-mm biopsy punch (Kai Corporation). Two PDMS layers were bonded to semipermeable PET membranes containing 3.0 μm pores (#353091, Corning) using a thin layer of liquid PDMS prepolymer as the mortar (2). PDMS prepolymer was spin-coated (4,000 rpm for 60 sec) onto a glass slide. Subsequently, both the top and bottom channel layers were placed on the glass slide to transfer the thin layer of PDMS prepolymer onto the embossed PDMS surfaces. The two PET membranes were placed on the top layer and then bonded to the bottom layer. The combined layers were left at room temperature for 1 day to remove air bubbles and then put into an oven at 60°C overnight to cure the PDMS glue. The PDMS-based microfluidic devices (PDMS devices) were sterilized by placing them under UV light for 1 hr prior to the cell culture.

SARS-CoV-2 preparation

The SARS-CoV-2 strains B.1.1.214 (EPI_ISL_2897162), B.1.617.2 (EPI_ISL_9636792), and B.1.1.529 (EPI_ISL_9638489) were isolated from nasopharyngeal swab samples of COVID-19 patients. This study has been approved by the research ethics committee of Kyoto University. SARS-CoV-2 B.1.1.214 was used for all figures other than **Figures S6B-S6D**. The virus was proliferated in TMPRSS2/Vero cells (JCRB1818, JCRB Cell Bank) and stored at -80°C (3). The cells were cultured with Minimum Essential Media (MEM, Sigma-Aldrich) supplemented with 5% FBS and 1% penicillin/streptomycin. All experiments including virus infections were done in a biosafety level 3 facility at Kyoto University strictly following regulations.

SARS-CoV-2 infection and drug treatment

SARS-CoV-2 (0.1 MOI) containing Hepatocyte Culture Medium BulletKit (HCM, Lonza) was injected into the top channel of ibd- and bv-LoCs. After 1 dpi (days post-infection), the culture medium was replaced with fresh medium and, the ibd- and bv-

LoCs was placed horizontally on an interval rocker switching (Infinity Rocker Mini, Next Advance), allowing bi-directional flow (Rocking rate of 0.2 cycles/min). In the drug treatment experiments, SARS-CoV-2-infected ibd- and bv-LoCs were treated with 2 μM remdesivir (RDV, A17170, Clinisciences), 10 μM molnupiravir (MPV, HY-135853, MedChemExpress), or 1 μM baricitinib (BARI, S2851, Selleck Chemicals).

Quantification of viral RNA copy number in the cell culture supernatant

The cell culture supernatant was mixed with an equal volume of 2×RNA lysis buffer (distilled water containing 0.4 U/μL SUPERase ITM RNase Inhibitor (Thermo Fisher Scientific), 2% Triton X-100, 50 mM KCl, 100 mM Tris-HCl (pH 7.4), and 40% glycerol) and incubated at room temperature for 10 min. The mixture was diluted 10 times with distilled water. Viral RNA was quantified using a One Step TB Green PrimeScript PLUS RT-PCR Kit (Perfect Real Time) (Takara Bio) on a QuantStudio 1 Real-Time PCR System (Thermo Fisher Scientific). The primers used in this experiment were as follows: (forward) AGCCTCTTCTCGTTCCTCATCAC and (reverse) CCGCCATTGCCAGCCATTC. Standard curves were prepared using SARS-CoV-2 RNA (10^5 copies/μL) purchased from Nihon Gene Research Laboratories.

Lactate dehydrogenase (LDH) release

To evaluate LDH release, the culture supernatants of the top and bottom channels of the ibd- and bv-LoCs were collected. The collected supernatants were analyzed using the LDH-Glo Cytotoxicity Assay (Promega) according to the manufacturer’s instructions.

Evaluation of transport activity of bile acid, bilirubin, and albumin

To evaluate the transport activity of bile acids, the culture supernatants of the top and bottom channels of the ibd- and bv-LoCs, which were cultured for 24 hr, were collected. The collected supernatants were analyzed using the Total Bile Acid Assay Kit (Cell Biolabs) according to the manufacturer’s instructions.

To evaluate the transport activity of direct bilirubin, 10 μM bilirubin was injected into the top channel. After 48 hr, the culture supernatants of the top and bottom channels were collected. The collected supernatants were analyzed using the QuantiChrom Bilirubin Assay Kit (BioAssay Systems) according to the manufacturer’s instructions.

To evaluate the transport activity of albumin, the culture supernatants of the top and bottom channels were collected at 4 days after the hepatocyte seeding. The collected supernatants were analyzed using the Human Albumin ELISA Quantitation Set (Bethyl Laboratories). ELISA was performed according to the manufacturer’s instructions.

RNA-seq analysis

RNA were isolated from human hepatocytes in infected ibd- and bv-LoCs. RNA integrity was assessed with a 2100 Bioanalyzer (Agilent Technologies). The library preparation was performed using a TruSeq stranded mRNA sample prep kit (Illumina) according to the manufacturer's instructions. Sequencing was performed on an Illumina NextSeq500. The fastq files were generated using bcl2fastq-2.20. Adapter sequences and low-quality bases were trimmed from the raw reads by Cutadapt v3.4 (4). The trimmed reads were mapped to the human reference genome sequences (hg38) using STAR v2.7.9a (5) with the GENCODE (release 36, GRCh38.p13) (6) gtf file. The raw counts for protein-coding genes were calculated using htseq-count v0.13.5 (7) with the GENCODE gtf file. Differentially expressed genes were identified with DEseq2 v1.30.1 (8), and gene expression levels were determined as transcripts per million (TPM). Raw data concerning this study was submitted under Gene Expression Omnibus (GEO) accession number GSE193330.

Oil Red O staining

Oil Red O stock solution was prepared by dissolving Oil Red O powder (Sigma-Aldrich) in 100% isopropanol to 3 mg/mL. Before staining, Oil Red O working solution was prepared by diluting the stock solution with distilled water (6:4). The ibd- and bv-LoCs were fixed with 4% paraformaldehyde in PBS for 15 min. After dehydration with 60% isopropanol for 1 min, the cells were treated with Oil Red O working solution for 1 hr.

BODIPY staining

The ibd- and bv-LoCs were fixed with 4% paraformaldehyde in PBS for 15 min and then incubated with 10 μg/mL BODIPY 493/503 (Thermo Fisher Scientific) for 30 min.

Cell viability assay in RDV-, MPV-, and BARI-treated hepatocytes

Before seeding human hepatocytes, a 96-well plate (Thermo Fisher Scientific) was pre-coated with Collagen Type I solution (30 μg/mL, Corning). Cryopreserved Human Hepatocytes (Lonza or Sekisui XenoTech) were used in this study. The vials of hepatocytes were rapidly thawed in a shaking water bath at 37°C; the contents of each vial were emptied into pre-warmed Cryopreserved Hepatocyte Recovery Medium (Thermo Fisher Scientific), and the suspension was centrifuged at 1200 rpm for 5 min at

room temperature. Then, the hepatocytes were seeded at 1.0x10^5 cells/cm^2 in HCM containing 10% FBS onto a Collagen I-coated 96-well plate. At 24 hr after seeding, the hepatocytes were treated with RDV, MPV, or BARI at several concentrations for 5 days. Cell viability was examined by the WST-8 assay using a Cell Counting Kit-8 (DOJINDO) according to the manufacturer's instructions. The cell viability was calculated as the percentage of that in cells treated with vehicle (DMSO) only.

Analysis of liver function markers in COVID-19 patients

Archived, residual serum samples from COVID-19 patients referred to Kyoto University Hospital were used in this study. These clinical specimens were previously archived for future studies to identify novel biomarkers for disease progression but were re-purposed for the present study. The severity of the patients was defined according to the criteria used by WHO guidance for the clinical management of COVID-19 released on 27 May 2020 (https://apps.who.int/iris/bitstream/handle/10665/332196/WHO-2019-nCoV-clinical-2020.5-eng.pdf?sequence=1&isAllowed=y). The reference ranges for normal values of each marker were as follows: AST, 13-30 U/L; ALT, 10-42 (male) and 7-27 (female) U/L; ALP, 38-113 U/L; and T-BIL, 0.4-1.5 mg/dL.

Quantification of viral RNA copy number in sera of COVID-19 patients

Viral RNA in serum samples from COVID-19 patients was quantified using a SARS-CoV-2 Direct Detection RT-qPCR Kit (Takara Bio) on a QuantStudio 5 Real-Time PCR System (Thermo Fisher Scientific).

Statistics

Statistical analyses were performed using GraphPad Prism8. Data are representative of three independent experiments. Details are described in the figure legends.

Study approval

The current study was performed in accordance with the Declaration of Helsinki and conducted with the approval of the Kyoto University Graduate School and Faculty of Medicine, Ethics Committee (R2379-3). Informed consent was obtained in the form of an opt-out form on the institution's website. The institutional ethics committee approved this informed consent plan.

Supplementary figures

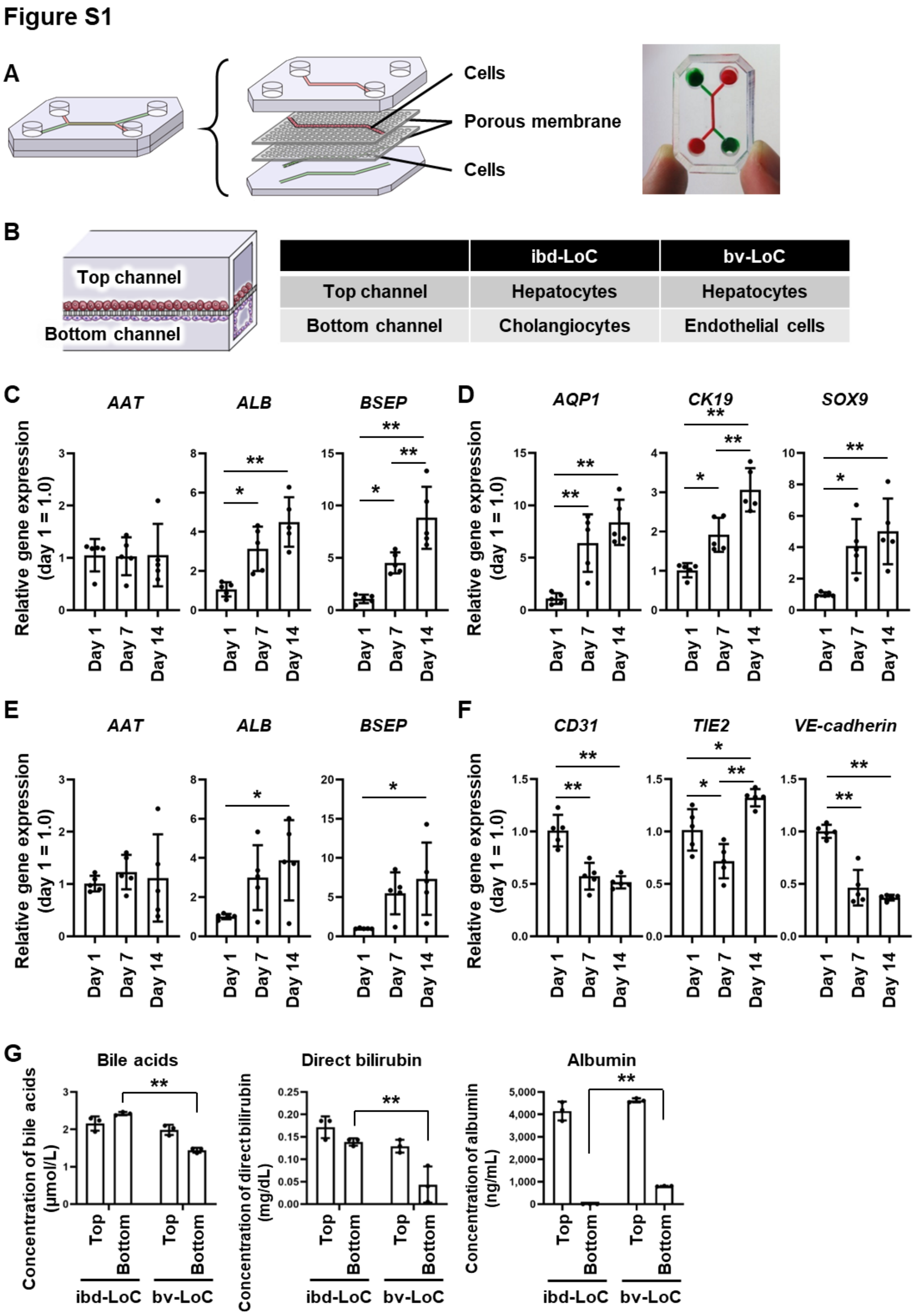

Figure S1 Generation of ibd- and bv-LoCs

(**A**) Schematic image of the microfluidic devices. (**B**) The cell components in the ibd- and

bv-LoCs. The ibd-LoCs were cultured for 1, 7, or 14 days, and (**C-D**) the expression levels of hepatocyte (*albumin* (*ALB*), *α1-antitrypsin* (*AAT*), and *bile salt export pump* (*BSEP*)) and cholangiocyte (*aquaporin 1* (*AQP1*), *cytokeratin19* (*CK19*), and *SRY-box transcription factor 9* (*SOX9*)) markers in hepatocytes (**C**) and cholangiocytes (**D**), respectively, in ibd-LoCs were examined by RT-qPCR analysis. One-way ANOVA followed by Tukey's post hoc test (*$p<0.05$, **$p<0.01$). Data are representative of three independent experiments and are represented as the means ± SD ($n=5$, technical replicates). The bv-LoCs were cultured for 1, 7, or 14 days, and (**E-F**) the expression levels of hepatocyte (*AAT*, *ALB*, and *BSEP*) and endothelial (*CD31*, *TIE2*, and *VE-cadherin*) markers in hepatocytes (**E**) and endothelial cells (**F**), respectively, in bv-LoCs were examined by RT-qPCR analysis. One-way ANOVA followed by Tukey's post hoc test (*$p<0.05$, **$p<0.01$). Data are representative of three independent experiments and are represented as the means ± SD ($n=5$, technical replicates). (**G**) The concentration of bile components (bile acids and direct bilirubin) or blood components (albumin) in the top and bottom channels of the ibd- and bv-LoCs. Two-way ANOVA with Bonferroni post hoc test (**$p<0.01$, ibd-LoC vs bv-LoC). Data are shown as means ± SD ($n=3$, technical replicates).

Figure S2

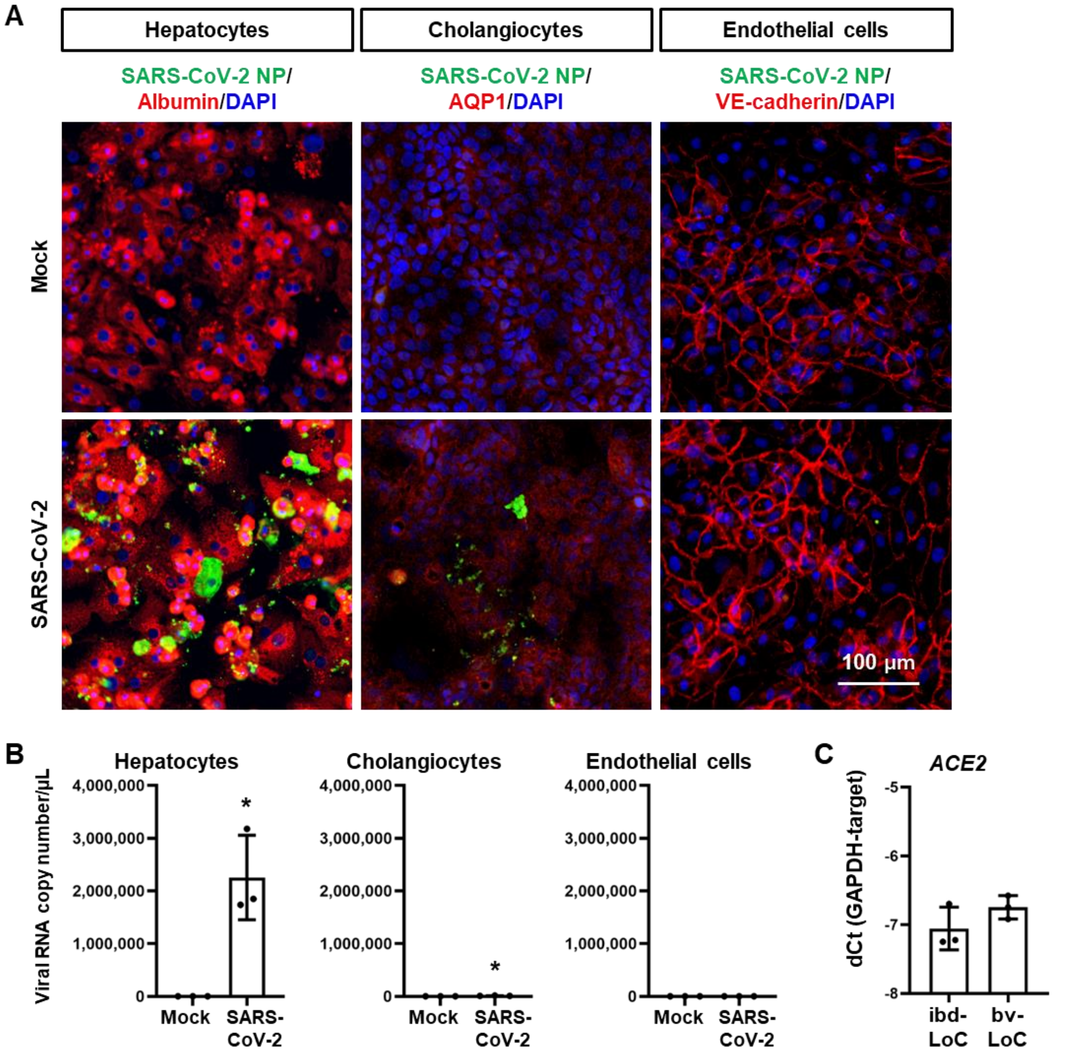

	Hepatocytes	Cholangiocytes	Endothelial cells
Mock (copies/μL)	Not detected	Not detected	Not detected
SARS-CoV-2 (copies/μL)	2,252,670±801,343	10,053±3,114	631±513

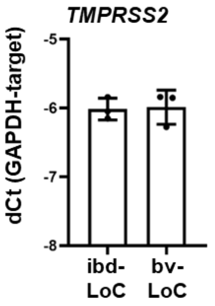

Figure S2 SARS-CoV-2 infects hepatocytes but not cholangiocytes or endothelial cells

Hepatocytes, cholangiocytes, or endothelial cells were cultured on a 96-well plate and infected with 0.1 MOI SARS-CoV-2. (**A**) At 4 dpi, immunostaining analysis was performed. Nuclei were counterstained with DAPI (blue). (**B**) At 4 dpi, the viral RNA

copy number in the cell culture supernatant was measured by qPCR. Unpaired two-tailed Student's *t*-test (*p<0.05). (**C**) The gene expression levels of *ACE2* and *TMPRSS2* in hepatocytes in ibd- and bv-LoCs were measured by RT-qPCR. Data are shown as means ± SD (n=3, technical replicates).

Figure S3

A

	2 dpi		7 dpi		14 dpi	
	Mock	SARS-CoV-2	Mock	SARS-CoV-2	Mock	SARS-CoV-2
ibd-LoC (copies/μL)	Not detected	104,179 ± 8,119	Not detected	1,800 ± 2503	Not detected	Not detected
bv-LoC (copies/μL)	Not detected	146,514 ± 47,972	Not detected	1,900 ± 90	Not detected	Not detected

B

	2 dpi		7 dpi		14 dpi	
	Mock	SARS-CoV-2	Mock	SARS-CoV-2	Mock	SARS-CoV-2
ibd-LoC (copies/μL)	Not detected	Not detected	Not detected	Not detected	Not detected	Not detected
bv-LoC (copies/μL)	Not detected	176,313 ± 32,898	Not detected	662 ± 189	Not detected	Not detected

Figure S3 SARS-CoV-2 infection efficiency of hepatocytes in ibd- and bv-LoCs
The ibd- and bv-LoCs were infected with 0.1 MOI SARS-CoV-2. At 2, 7, and 14 dpi, the viral RNA copy number in the cell culture supernatant in the top channel (**A**) or bottom channel (**B**) was measured by qPCR. Data are shown as means ± SD (*n*=3, technical replicates).

Figure S4

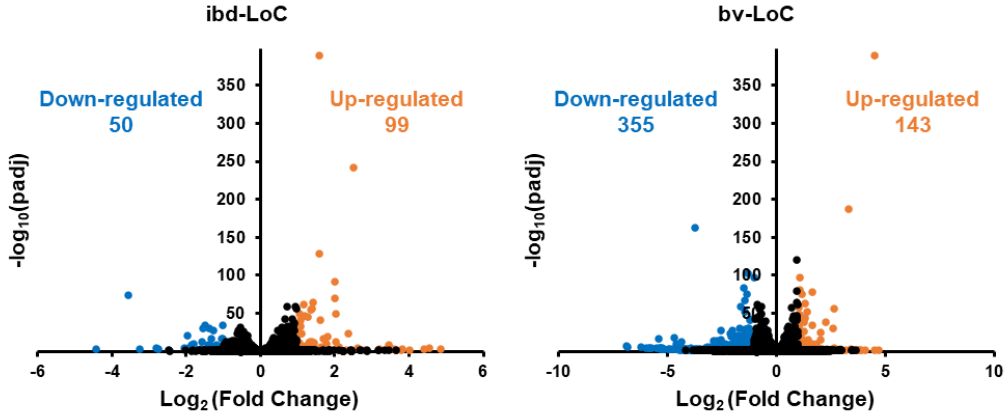

Figure S4 RNA-seq analysis of hepatocytes in SARS-CoV-2-infected ibd- and bv-LoCs

The ibd-LoCs (left) and bv-LoCs (right) were infected with 0.1 MOI SARS-CoV-2. RNA-seq analysis of mock- or SARS-CoV-2-infected hepatocytes in ibd- and bv-LoCs at 4 dpi. A volcano plot of differentially expressed genes between mock and infected hepatocytes ($\log_2$ fold-change > 2, adjusted *p*-value (padj) < 0.01). Orange dots represent up-regulated genes and blue dots represent down-regulated genes.

Figure S5

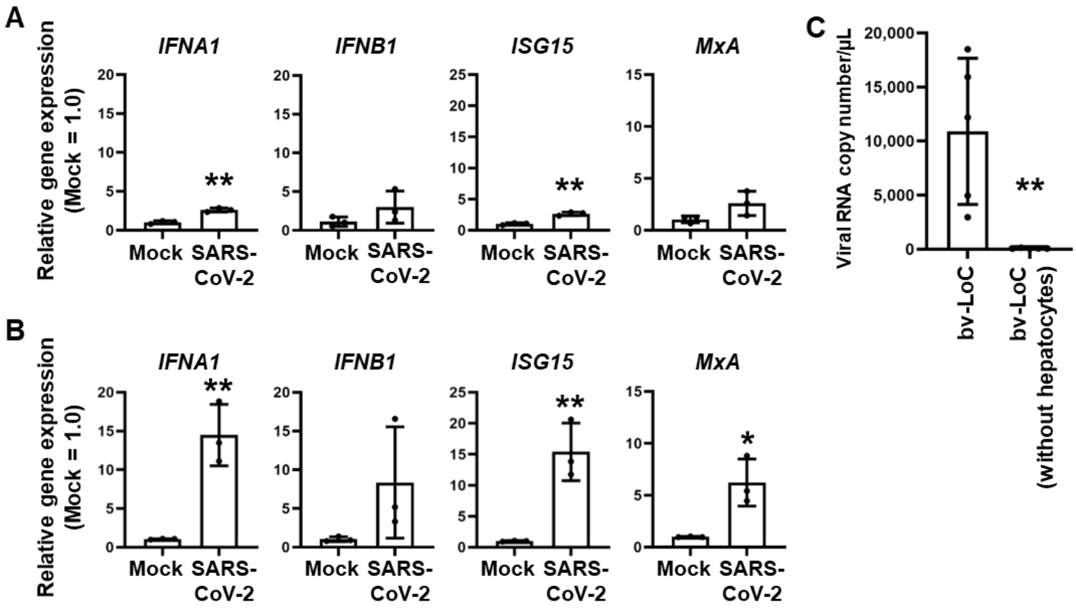

Figure S5 The gene expression levels of IFN-related markers were increased by SARS-CoV-2 infection in hepatocytes of bv-LoCs

The ibd- and bv-LoCs were infected with 0.1 MOI SARS-CoV-2. (**A-B**) The gene expression levels of IFN-related markers (*IFNA1*, *IFNB1*, *ISG15*, and *MxA*) in cholangiocytes in ibd-LoCs (**A**) or endothelial cells in bv-LoCs (**B**) were measured by RT-qPCR. The gene expression levels in mock were taken as 1.0. Unpaired two-tailed Student's *t*-test (**p*<0.05, ***p*<0.01). Data are shown as means ± SD (*n*=3, technical replicates). (**C**) At 2 dpi, the viral RNA copy number in the cell culture supernatant in the bottom channel of bv-LoC was measured by qPCR in the presence or absence of hepatocytes. Unpaired two-tailed Student's *t*-test (***p*<0.01). Data are shown as means ± SD (*n*=5, technical replicates).

Figure S6

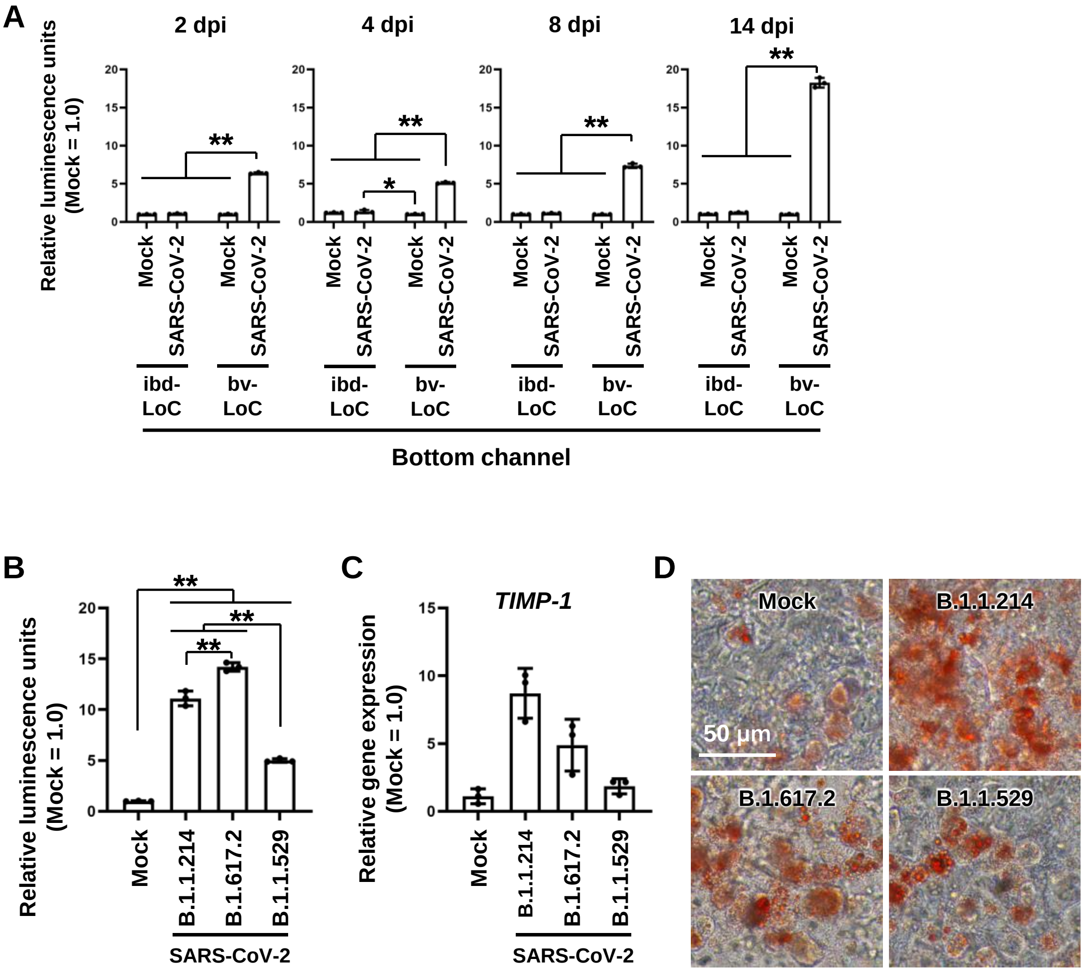

Figure S6 Liver damage was caused in SARS-CoV-2 infected hepatocytes in bv-LoCs

The ibd- and bv-LoCs were infected with 0.1 MOI SARS-CoV-2. (**A**) At 2, 4, 8, and 14 dpi, LDH release in the cell culture supernatant was measured. The LDH release in mock was taken as 1.0. Two-way ANOVA followed by Tukey's post hoc test (*p<0.05, **p<0.01). (**B-D**) bv-LoCs were infected with 0.1 MOI SARS-CoV-2 (B.1.1.214, B.1.617.2, or B.1.1.529). (**B**) At 2 dpi, LDH release in the cell culture supernatant in the top channel was measured. The LDH release in mock was taken as 1.0. Two-way ANOVA followed by Tukey's post hoc test (**p<0.01). (**C**) At 14 dpi, the gene expression levels of *TIMP1* were measured by RT-qPCR. The gene expression levels in mock were taken as 1.0. (**D**) At 14 dpi, lipid droplets in hepatocytes in ibd- and bv-LoCs were stained with Oil red O. Data are representative of three independent experiments and are represented as the means ± SD (n=3, technical replicates).

Figure S7

A

	DiffCoef	MlogP	S+logP	S+logD	MWt	tPSA
RDV	0.512	0.634	1.597	1.597	602.588	203.55
MPV	0.76	-0.456	-1.315	-1.324	329.312	143.14
BARI	0.697	-0.426	0.33	0.33	371.423	120.56

B

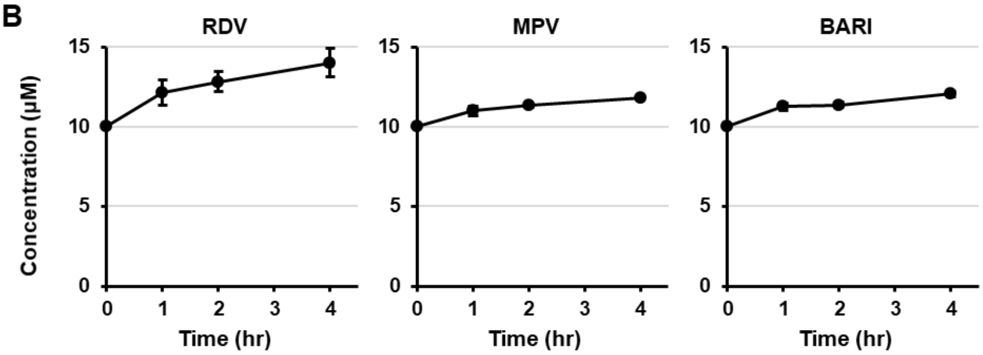

Figure S7 Absorption of RDV, MPV, or BARI by the PDMS device

(**A**) Physicochemical properties (diffusion coefficient (DiffCoef), partition coefficients (MLogP, S+LogP, and S+logD), molecular weight (MWt), and topological polar surface area (tPSA)) of RDV and MPV were calculated using MedChem Designer 5.5. (**B**) HBSS containing RDV, MPV, or BARI was injected into the PDMS device. At 1, 2, and 4 hr after the injection, the quantity of these drugs was measured. Data are shown as means ± SD (*n*=3, technical replicates).

Figure S8

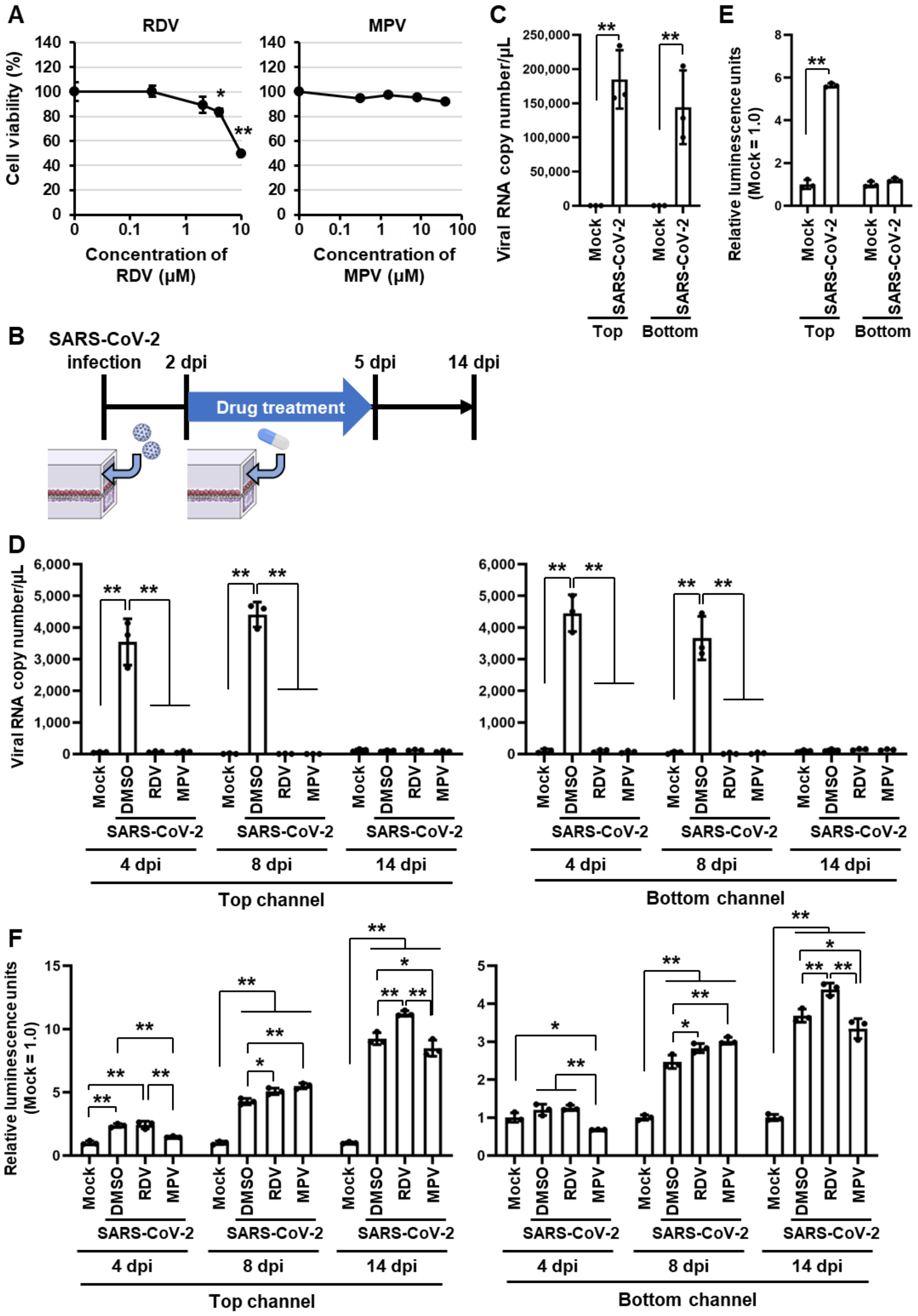

Figure S8 Effects of RDV or MPV treatments in the infected bv-LoC

(**A**) Hepatocytes were seeded on a 96-well plate and cultured with medium containing RDV or MPV for 5 days. Cell viability was measured by the WST-8 assay. Unpaired two-tailed Student's *t*-test (**p*<0.05, ***p*<0.01). (**B**) Schematic overview showing the protocol for the SARS-CoV-2 infection and treatment with RDV or MPV. The bv-LoC was

infected with 0.1 MOI SARS-CoV-2. At 2 dpi, the medium containing RDV or MPV was injected into the top channel of the bv-LoC. Three days after the drug treatment, the medium was replaced with a fresh medium. (**C**) At 2 dpi, the viral RNA copy number in the cell culture supernatant was measured by qPCR. Two-way ANOVA with Bonferroni post hoc test (**p<0.01, Mock vs SARS-CoV-2). (**D**) At 4, 8, and 14 dpi, the viral RNA copy number in the cell culture supernatant was measured by qPCR. Two-way ANOVA followed by Tukey's post hoc test (**p<0.01). (**E**) At 2 dpi, LDH release in the cell culture supernatant was measured. Two-way ANOVA with Bonferroni post hoc test (**p<0.01, Mock vs SARS-CoV-2). (**F**) At 4, 8, and 14 dpi, LDH release in the cell culture supernatant in the bottom channel was measured. Two-way ANOVA followed by Tukey's post hoc test (*p<0.05, **p<0.01). Data are shown as means ± SD (n=3, technical replicates).

Figure S9

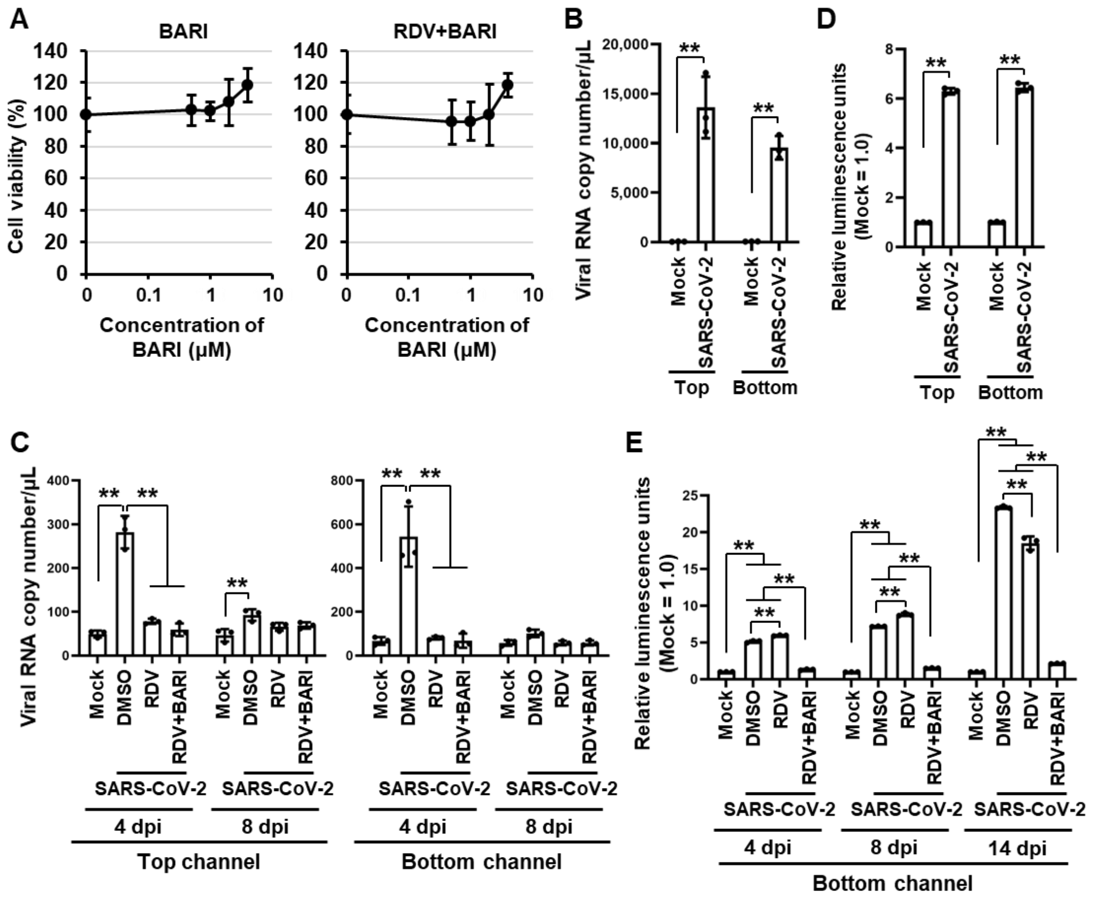

Figure S9 Effects of the combination of RDV and BARI

(**A**) Hepatocytes were seeded on a 96-well plate and cultured with medium containing RDV only or both RDV and BARI for 5 days. Cell viability was measured by the WST-8 assay. (**B**) At 2 dpi, the viral RNA copy number in the cell culture supernatant was measured by qPCR. Two-way ANOVA with Bonferroni post hoc test (**p<0.01, Mock vs SARS-CoV-2). (**C**) At 4 and 8 dpi, the viral RNA copy number in the cell culture supernatant was measured by qPCR. Two-way ANOVA followed by Tukey's post hoc test (**p<0.01). (**D**) At 2 dpi, LDH release in the cell culture supernatant was measured. Two-way ANOVA with Bonferroni post hoc test (**p<0.01, Mock vs SARS-CoV-2). (**E**) At 4, 8, and 14 dpi, LDH release in the cell culture supernatant in the bottom channel was measured. Two-way ANOVA followed by Tukey's post hoc test (**p<0.01). Data are shown as means ± SD (n=3, technical replicates).

Figure S10

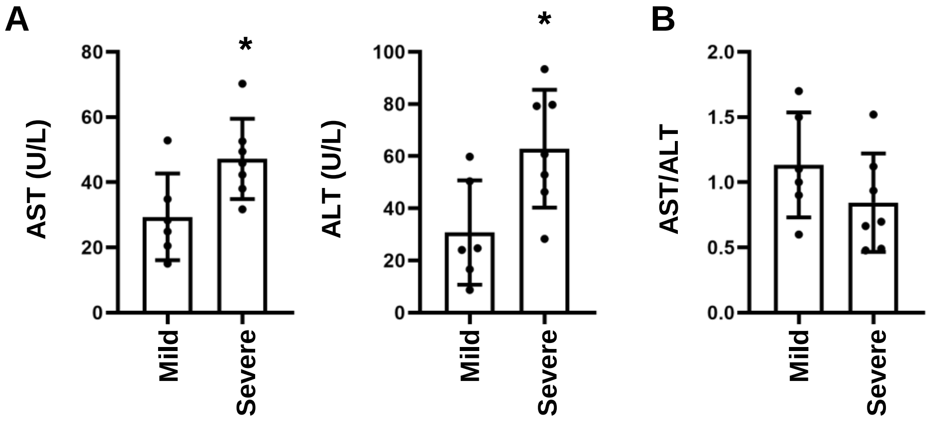

Figure S10 Serum AST and ALT values in mild and severe COVID-19 patients
(**A**) The average values of AST and ALT in patients with mild (*n*=6) and severe COVID-19 (*n*=7) (1-14 days after the onset). Unpaired two-tailed Student's *t*-test (*$p<0.05$). (**B**) The absolute AST/ALT values in patients with mild and severe COVID-19 (1-14 days after the onset). Data are shown as means ± SD (technical replicates).

Table S1 Analysis of liver function markers and viral RNA in serum obtained from COVID-19 patients

patient	1	2	3	4	5	6	7	8
Sex	female	female	male	female	male	male	male	male
Severity	Mild	Mild	Mild	Moderate	Moderate	Moderate	Moderate	Moderate
AST (1-7 days) (U/L)	22	21	12	26	23	56.8	35	47.3
ALT (1-7 days) (U/L)	19	9.3	15.5	23	25	45.8	29	47.3
serum virus (copies/mL)	N.D.	N.D.	88.8	N.D.	N.D.	N.D.	81.3	281.0

patient	9	10	11	12	13	14	15
Sex	male	male	female	female	male	male	male
Severity	severe	severe	severe	severe	severe	severe	severe
AST (1-7 days) (U/L)	47	87.3	39	98.8	45.6	73.4	13.3
ALT (1-7 days) (U/L)	49.6	69.7	27.3	-	43.4	63.6	5.3
serum virus (copies/mL)	270.1	364.5	1782.9	3574.2	918.7	405.5	287.4

Table S2 Primers used in the qPCR analysis

Gene Symbol	Primers (forward/reverse; 5' to 3')
Claudin-1	AGTGCTTGGAAGACGATGAGG/ACTGGGGTCATAGGGTCATAG
Occludin	GGCAAAGTGAATGACAAGCG/CACAGGCGAAGTTAATGGAAGC
GJA5	CCGTGGTAGGCAAGGTCTG/ATCACACCGGAAATCAGCCTG
COL1A1	GAGGGCCAAGACGAAGACATC/CAGATCACGTCATCGCACAAC
TIMP1	CTTCTGCAATTCCGACCTCGT/ACGCTGGTATAAGGTGGTCTG
IFNA1	GCCTCGCCCTTTGCTTTACT/CTGTGGGTCTCAGGGAGATCA
IFNB1	ATGACCAACAAGTGTCTCCTCC/GGAATCCAAGCAAGTTGTAGCTC
ISG15	GCAGATCACCCAGAAGATCG/GGCCCTTGTTATTCCTCACC
MxA	CTTATCCGTTAGCCGTGGTG/CAAGGTGGAGCGATTCTGAG
GAPDH	GGTGGTCTCCTCTGACTTCAACA/GTGGTCGTTGAGGGCAATG
AQP1	ATTAACCCTGCTCGGTCCTT/ACCCTGGAGTTGATGTCGTC
CK19	CTCCCGCGACTACAGCCACT/TCAGCTCATCCAGCACCCTG
SOX9	GTACCCGCACTTGCACAAC/TCTCGCTCTCGTTCAGAAGTC
CD31	AACAGTGTTGACATGAAGAGCC/TGTAAAACAGCACGTCATCCTT
TIE2	TACACCTGCCTCATGCTCAG/TTCACAAGCCTTCTCACACG
VE-cadherin	TTGGAACCAGATGCACATTGAT/TCTTGCGACTCACGCTTGAC
ALB	TGCAACTCTTCGTGAAACCTATG/ACATCAACCTCTGGTCTCACC
AAT	ACTGTCAACTTCGGGGACAC/CATGCCTAAACGCTTCATCA
BSEP	TTGGCTGATGTTTGTGGGAAG/CCAAAAATGAGTAGCACGCCT

Table S3 Antibodies used in the immunofluorescence staining

Antibody	Company	Catalogue #
Albumin	Bethyl Laboratories	A80-229A
CK19	Abcam	ab52625
CD31	Proteintech	11265-1-AP
SARS-CoV-2 NP	BIO Vision	A2061-50
AQP1	Santa Cruz Biotechnology	sc-25287
VE-cadherin	BD Pharmingen	555661
Donkey anti-Goat IgG (H+L) Cross-Adsorbed Secondary Antibody, Alexa Fluor 488	Thermo Fisher Scientific	A-11055
Donkey anti-Rabbit IgG (H+L) Highly Cross-Adsorbed Secondary Antibody, Alexa Fluor 488	Thermo Fisher Scientific	A-21206
Chicken anti-Rabbit IgG (H+L) Cross-Adsorbed Secondary Antibody, Alexa Fluor 594	Thermo Fisher Scientific	A-21442
Chicken anti-Mouse IgG (H+L) Cross-Adsorbed Secondary Antibody, Alexa Fluor 594	Thermo Fisher Scientific	A-21201

Table S4 HPLC methods

Remdesivir (RDV)	
Column	COSMOSIL(R) 5C18-AR-Ⅱ<br>Packed Column 4.6 mm I.D. x 150 mm (#38144-31, Nacalai Tesque)
Mobile phase (A)	20 mM Phosphate buffer (pH 3.0)
Mobile phase (B)	Acetonitrile
Flow-rate (ml/min)	1 mL/min A:B = 55:45, v/v
Temperature	30 ℃
Detection	Fluorescence 245 nm / 390 nm
Retention time	5.7 min

Molnupiravir (MPV)	
Column	COSMOSIL(R) 5C18-MS-II<br>Packed Column 4.6 mm I.D. x 150 mm (#38019-81, Nacalai Tesque)
Mobile phase (A)	Water
Mobile phase (B)	Acetonitrile
Flow-rate (ml/min)	1 mL/min A:B = 85:15, v/v
Temperature	40 ℃
Detection	UV 235 nm
Retention time	6.2 min

Baricitinib (BARI)	
Column	COSMOSIL(R) 5C18-MS-II<br>Packed Column 4.6 mm I.D. x 150 mm (#38019-81, Nacalai Tesque)
Mobile phase (A)	10 mM Phosphate buffer (pH 7.4)
Mobile phase (B)	Acetonitrile
Flow rate (ml/min)	1 mL/min A:B = 75:25, v/v
Temperature	40 ℃

Detection	UV 251 nm
Retention time	5.6 min

Supplementary references

1. D. C. Duffy, J. C. McDonald, O. J. A. Schueller, G. M. Whitesides, Rapid prototyping of microfluidic systems in poly(dimethylsiloxane). *Analytical Chemistry* **70**, 4974-4984 (1998).
2. B. H. Chueh *et al.*, Leakage-free bonding of porous membranes into layered microfluidic array systems. *Analytical Chemistry* **79**, 3504-3508 (2007).
3. S. Matsuyama *et al.*, Enhanced isolation of SARS-CoV-2 by TMPRSS2-expressing cells. *Proceedings of the National Academy of Sciences of the United States of America* **117**, 7001-7003 (2020).
4. M. Martin, Cutadapt removes adapter sequences from high-throughput sequencing reads. *EMBnet. journal* **17**, 10-12 (2011).
5. A. Dobin *et al.*, STAR: ultrafast universal RNA-seq aligner. *Bioinformatics* **29**, 15-21 (2013).
6. A. Frankish *et al.*, GENCODE reference annotation for the human and mouse genomes. *Nucleic Acids Research* **47**, D766-D773 (2019).
7. S. Anders, P. T. Pyl, W. Huber, HTSeq-a Python framework to work with high-throughput sequencing data. *Bioinformatics* **31**, 166-169 (2015).
8. M. I. Love, W. Huber, S. Anders, Moderated estimation of fold change and dispersion for RNA-seq data with DESeq2. *Genome Biology* **15**, 1-21 (2014).
9. Y. Chen *et al.*, A versatile polypharmacology platform promotes cytoprotection and viability of human pluripotent and differentiated cells. *Nature Methods* **18**, 528-541 (2021).
